# Supplementary material for: Sensitivity enhancement of homonuclear multidimensional NMR correlations for labile sites in proteins, polysaccharides, and nucleic acids
Source: Nat Commun. 2020 Oct 21;11:5317. doi: 10.1038/s41467-020-19108-x (PMC7577996; doi:10.1038/s41467-020-19108-x)
Supplement: Supplementary file 1 — Supplementary Information [file 41467_2020_19108_MOESM1_ESM.pdf]

Supplementary Information for

Sensitivity enhancement of homonuclear multidimensional NMR correlations for labile sites in proteins, polysaccharides and nucleic acids

Mihajlo Novakovic[1], Ēriks Kupče[2], Andreas Oxenfarth[3], Marcos D. Battistel[4], Darón I. Freedberg[4], Harald Schwalbe[3] and Lucio Frydman[1,*]

[1]Department of Chemical and Biological Physics, Weizmann Institute of Science, 7610001 Rehovot, Israel

[2]Bruker UK Ltd., Banner Lane, Coventry, UK

[3]Institute for Organic Chemistry and Chemical Biology, Center for Biomolecular Magnetic Resonance, Johann Wolfgang Goethe-University, D-60438 Frankfurt/Main, Germany

[4]Laboratory of Bacterial Polysaccharides, Center for Biologics Evaluation and Research, Food and Drug Administration, 10903 New Hampshire Ave, Silver Spring, Maryland, United States 20993

[*]To whom correspondence may be addressed: lucio.frydman@weizmann.ac.il

A theoretical analysis of HMT's signal enhancement.

To describe and quantify HMT, the process was assessed using a Bloch-McConnell-Solomon model[1–4] based on coupled differential equations that follow the fate of magnetization upon selective manipulation of the labile spins' pool. These labile protons were allowed to undergo suitably-population-weighted chemical exchanges with the solvent (water), while connected to a non-labile spin pool receiving polarization via a generic cross-relaxation[5] or J-coupling[6] process represented by a rate σ. The resulting relevant equations can be written as:

$$\begin{aligned}
\frac{dM_{y_l}}{dt} &= \omega_1 M_{z_l} - \left(R_{2_l} + k_{ex}^l\right) M_{y_l} + k_{ex}^w M_{y_w} \\
\frac{dM_{z_l}}{dt} &= -\omega_1 M_{y_l} - \left(R_{1_l} + k_{ex}^l + \sigma\right) M_{z_l} + \sigma M_{z_{nl}} + k_{ex}^w M_{z_w} + R_{1_l} M_{z_l}^0 \\
\frac{dM_{z_{nl}}}{dt} &= -\left(R_{1_{nl}} + \sigma\right) M_{z_{nl}} + \sigma M_{z_l} + R_{1_{nl}} M_{z_{nl}}^0 \\
\frac{dM_{z_w}}{dt} &= -\left(R_{1_w} + k_{ex}^w\right) M_{z_w} + k_{ex}^l M_{z_l} + R_{1_w} M_{z_w}^0 \\
\frac{dM_{y_w}}{dt} &= -\left(R_{2_w} + k_{ex}^w\right) M_{y_w} + k_{ex}^l M_{y_l}
\end{aligned} \tag{1}$$

where M_{y_l}, M_{z_l}, $M_{z_{nl}}$, M_{z_w}, M_{y_w} are the magnetization components of the labile, non-labile and water spin reservoirs along specified axis of the Bloch sphere, and $M_{z_l}^0$, M_{znl}^0, $M_{z_w}^0$ correspond to equilibrium magnetizations of these reservoirs (assumed for simplicity normalized to unity). Longitudinal and transverse relaxation rates are accounted as the inverse of the corresponding relaxation times $R_{1(2)} = 1/T_{1(2)}$ for each species, and, in order to account for population differences between the solute and water pools, the exchange rates of the labile and water ^{1}Hs are scaled according to:

$$k_{ex}^l[solute] = k_{ex}^w[water] \tag{2}$$

For the specific instance of a NOESY-based HMT experiment, σ represents the difference between zero- and double-quantum dipole-dipole cross-relaxation rates, and can be expressed in terms of normalized spectral densities $\mathcal{J}$ as

$$\sigma = \frac{1}{10} b^2 \left(\mathcal{J}(0) - 6\mathcal{J}(2\omega^0) \right) \tag{3}$$

where $\mathcal{J}(\omega) = \frac{\tau_c}{1+\omega^2 \tau_c^2}$ and $b = -\frac{\mu_0}{4\pi} \frac{\hbar\gamma^2}{r^3}$ is the dipole-dipole coupling constant.[7]

The steady-state (ss) solutions of Supplementary Eq. (1) can be derived by setting all derivatives to zero. The change of magnetization of the non-labile spin pool upon perturbation of labile proton; *i.e.*, the cross-relaxation-driven HMT "cross peak", can then be expressed as

$$m_{z_{nl}}^{MT_{ss}} = M_{z_{nl}}^{MT_{ss}} - M_{z_{nl}}^{0} =$$

$$= \frac{{\nu_1}^2 \sigma M_{z_l}^0}{\left\{ R_{2_l} + k_{ex}^l - \frac{k_{ex}^l * k_{ex}^w}{R_{2_w} + k_{ex}^w} \right\} \left\{ \left(R_{1_l} + k_{ex}^l + \sigma - \frac{k_{ex}^l * k_{ex}^w}{R_{1_w} + k_{ex}^w} \right) (R_{1_{nl}} + \sigma) - \sigma^2 \right\} + {\nu_1}^2 (R_{1_{nl}} + \sigma)} \tag{4}$$

This expression can be simplified by introducing the substitutions $p = \left(R_{2_l} + k_{ex}^l - \frac{k_{ex}^l * k_{ex}^w}{R_{2_w} + k_{ex}^w} \right)$ and $q = \left(R_{1_l} + k_{ex}^l + \sigma - \frac{k_{ex}^l * k_{ex}^w}{R_{1_w} + k_{ex}^w} \right) (R_{1_{nl}} + \sigma) - \sigma^2$, leading to:

$$m_{z_{nl}}^{MT_{ss}} = \frac{{\nu_1}^2 \sigma}{pq + {\nu_1}^2 (R_{1_{nl}} + \sigma)} M_{z_l}^0 = a_{l \to nl}^{MT_{ss}} M_{z_l}^0 \tag{5}$$

In order to examine the HMT enhancement with respect to a conventional NOESY experiment, similar exchange-edited Solomon's equations were used to analytically calculate the cross-peak buildup for the latter experiment. A simplification was introduced by exploiting the fact that the water spin pool is much larger than either labile or non-labile spin pools; water chemical exchange can thus be considered simply as sources of relaxation/repolarization for the labile proton, enabling one to employ a two-site Solomon equation in order to describe the matrix driving the NOESY experiment:

$$R = \begin{pmatrix} -\rho_1 & \sigma \\ \sigma & -\rho_2 \end{pmatrix} = \begin{pmatrix} -(R_{1_l} + k_{ex}^l + \sigma) & \sigma \\ \sigma & -(R_{1_{nl}} + \sigma) \end{pmatrix} \tag{6}$$

This matrix R can be easily diagonalized and, after finding its eigenvalues $\lambda_\pm$, the cross-terms driving exchanges between the labile and non-labile spin pools can be calculated and expressed as:

$$a_{l \to nl}(t) = a_{nl \to l}(t) = \frac{\sigma}{\lambda_+ - \lambda_-} \left(\mathrm{e}^{-\lambda_- t} - \mathrm{e}^{-\lambda_+ t} \right) \tag{7}$$

where $\lambda_\pm = \frac{1}{2} \left\{ (R_{1_l} + R_{1_{nl}} + 2\sigma + k_{ex}^l) \pm \sqrt{(R_{1_l} + k_{ex}^l - R_{1_{nl}})^2 + 4\sigma^2} \right\}$. The maximum value of this cross-transfer process can be found by differentiating Supplementary Eq. (7); this leads to the extremum of $a_{l \to nl}(t)$ and, from there, to the optimal mixing time for maximizing cross-peaks in the NOESY experiment: $t_{opt} = \frac{\ln \frac{\lambda_+}{\lambda_-}}{\lambda_+ - \lambda_-}$. When substituted in Supplementary Eq. (7), this t_{opt} leads to the maximum achievable NOE cross-peak intensity:

$$a_{l\to nl}^{NOESY_{max}} = a_{nl\to l}^{NOESY_{max}} = \frac{\sigma}{\lambda_+ - \lambda_-}\left(\mathrm{e}^{-\lambda_- \frac{\ln\frac{\lambda_+}{\lambda_-}}{\lambda_+ - \lambda_-}} - \mathrm{e}^{-\lambda_+ \frac{\ln\frac{\lambda_+}{\lambda_-}}{\lambda_+ - \lambda_-}} \right) \quad (8)$$

Notice that unlike what was derived for HMT, where the cross-peak intensity will be mostly constrained by the receiving spin pool's $T_{1_{nl}}$, NOESY cross peaks will depend on the effective relaxation of both spin pools. Notice as well that intensity-wise they will be symmetrical with respect to the diagonal peaks, whereas in HMT they would be not. In the fast exchanging limit, when $k_{ex}^{l} \gg R_{1_l}, R_{1_{nl}}, \sigma$, Supplementary Equations (5) and (8) can be further simplified to

$$\begin{gathered} a_{l\to nl}^{MT_{ss}} = \frac{{\omega_1}^2 \sigma}{({k_{ex}^{l}}^2 + {\omega_1}^2)(R_{1_{nl}} + \sigma)} \\ a_{l\to nl}^{NOESY_{max}} = \frac{\sigma}{k_{ex}^{l}} \end{gathered} \quad (9)$$

One can then define a relative enhancement factor, ε, between the labile→non-labile cross-peak intensities in the HMT and NOESY experiments, that will be given by:

$$\varepsilon = \frac{a_{l\to nl}^{MT_{ss}}}{a_{l\to nl}^{NOESY_{max}}} \approx \frac{{\nu_1}^2 k_{ex}^{l} T_{1_{nl}}^{eff}}{({k_{ex}^{l}}^2 + {\nu_1}^2)} \quad (10)$$

This is Eq. (3) presented in the main text, which as further analyzed there explains the CEST-like gains that under suitable conditions will benefit the HMT experiments over their conventional 2D counterparts.

The analytical derivation above relies on a continuous ν_1 saturation radiofrequency field, assumed applied along the *x*-axis. As mentioned, HMT can also be achieved using looped inversion pulses. To explore the performance of this experimental implementation analytical derivations were replaced by numerical simulations based on a similar model as given in Supplementary Eq. (1), but with the transverse saturation terms omitted. This left equations that only depend on the M_z magnetization components:

$$\begin{gathered} \frac{dM_{z_l}}{dt} = -\left(R_{1_l} + k_{ex}^{l} + \sigma\right)M_{z_l} + \sigma M_{z_{nl}} + k_{ex}^{w} M_{z_w} + R_{1_l} M_{z_l}^{0} \\ \frac{dM_{z_{nl}}}{dt} = -\left(R_{1_{nl}} + \sigma\right)M_{z_{nl}} + \sigma M_{z_l} + R_{1_{nl}} M_{z\_nl}^{0} \\ \frac{dM_{z_w}}{dt} = -\left(R_{1_w} + k_{ex}^{w}\right)M_{z_w} + k_{ex}^{l} M_{z_l} + R_{1_w} M_{z_w}^{0} \end{gathered} \quad (11)$$

To account for the selective inversions of the labile [1]Hs an initial magnetization $M_z(0) = \begin{pmatrix} -1 \\ 1 \\ 1 \end{pmatrix}$ was taken as starting point, and numerical propagations were repeated l_1 times assuming that after propagating Supplementary Eq. (11) for a period τ_{mix}, a perturbation (i.e., the selective inversion) transformed $M_z(i \cdot \tau_{mix}) \longrightarrow UM_z(i \cdot \tau_{mix})$, where $U = \begin{pmatrix} -1 & 0 & 0 \\ 0 & 1 & 0 \\ 0 & 0 & 1 \end{pmatrix}$ represents the selective labile proton inversion, and $i = 1, \dots, l_1 - 1$.

Supplementary Figure 1 shows the fate of non-labile magnetization subject to these manipulations, summarizing the cross-peak intensities expected from numerical simulations

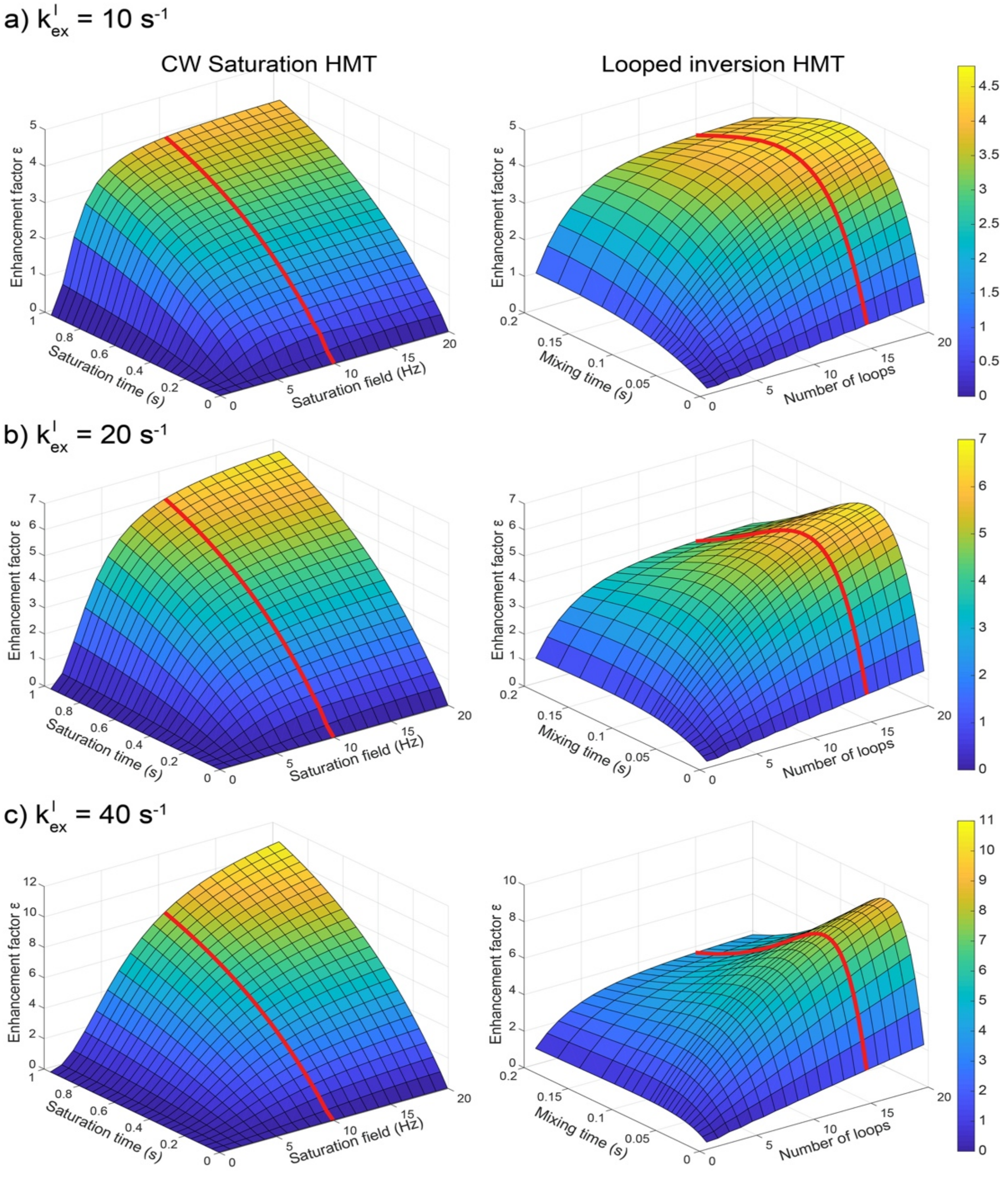

Supplementary Figure 1. Enhancement factors ε calculated for CW saturation and for looped inversion versions of HMT-based experiments vs conventional NOESY transfers, computed for different rates of chemical exchange with the solvent. Cross-relaxation rates were calculated at 14.1 T for $\tau_c = 0.1\ ns$ correlation times and $r = 2$ Å internuclear distance. Labile and non-labile sites relaxation rates were chosen as $T_{2_l} = T_{2_{nl}} = 0.3\ s$, $T_{1_l} = 0.5\ s$ and $T_{1_{nl}} = 0.8\ s$; water relaxation constants were taken $T_{2_w} = 0.5\ s$ and $T_{1_w} = 3\ s$. A water excess of 500-fold was assumed. Red curves illustrate the conditions that in terms of saturation fields and number of loops, were usually explored in this study's experiments.

for the two forms of HMT-related procedures introduced in this study. To better quantify these effects, the *z*-axes of these 3D plots are plotted as enhancements vs conventional NOESY cross-peak intensities derived by solutions of Supplementary Eq. (6), and calculated

numerically as $\varepsilon = M_{z_{nl}}(\tau_{sat})/M_{z_{nl}}(\tau_{mix}^{opt}, l_1 = 1)$ for a saturation-based HMT, and as $\varepsilon = M_{z_{nl}}(\tau_{mix,}l_1)/M_{z_{nl}}(\tau_{mix}^{opt}, l_1 = 1)$ for the looped inversion HMT. These enhancements are plotted vs the main parameters of these experiments: saturation time and nutation field ν_l for the continuous irradiation case (left), and number of loops and mixing time per loop for the repeated inversion experiment (column). A small molecule scenario with fast tumbling ($\tau_c = 0.1$ ns) and short internuclear distances ($r = 2$ Å) was used in the cross-relaxation rate calculations, and different chemical exchange rates k_{ex}^l with the solvent were examined. In the case of a slower chemical exchange the expected enhancements reach factors ≤5, while for faster exchange rates HMT boosts cross-peak intensities by an order of magnitude vs conventional NOESY. This is understandable since, as long as they take place in the slow- to mid-rate exchange regime (i.e., as long as solvent and solute lines are well separated), faster chemical exchanges will detract from conventional cross-relaxation transfers but enhance HMT's efficiency by providing a more rapid and complete repolarization of the labile site from the abundant water pool. Chemical exchange rates also affect other aspects of the HMT enhancement: For slow k_{ex}^l even low ν_l fields suffice to provide efficient saturation of the labile protons and high enhancements (Supplementary Fig. 1a), while the enhancements need and benefit from higher ω_1s when the repolarization by chemical exchange becomes fast (Supplementary Fig. 1c). Furthermore, when relying on looped inversions, faster chemical exchange repolarize labile ^{1}Hs more quickly, shifting the maximal transfer enhancements towards shorter mixing times and higher numbers of loops. Additional features including dependencies of HMT enhancements on correlation times, internuclear distances and chemical exchange rates are summarized further in Supplementary Figures 2 and 3. Predictions of a similar model focusing on the enhancements anticipated for *J*-based HMT TOCSY transfers, are presented in Supplementary Figure 4.

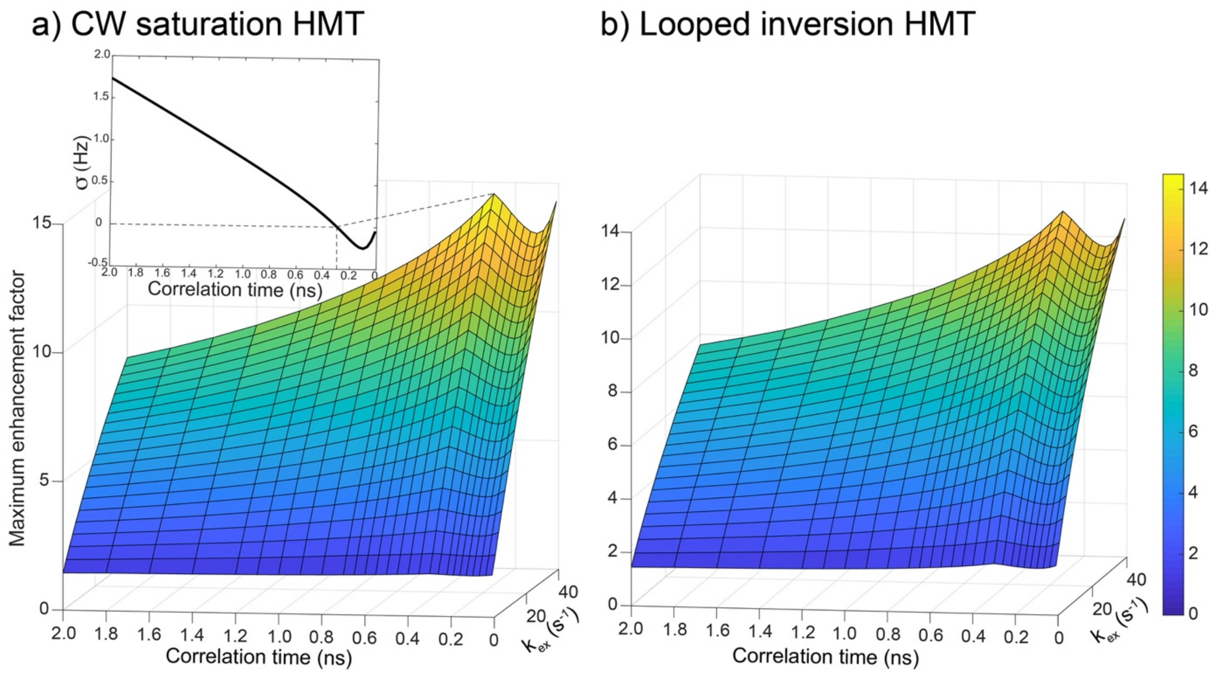

Supplementary Figure 2. Maximum achievable enhancements of Hadamard MT NOESY with respect to correlation times and chemical exchange rates. (a) Saturation-based implementation. The non-monotonic change shown by the enhancement with respect to correlation times can be explained by the non-monotonic cross-relaxation rates' dependence on the τ_c, as shown in the inset. (b) Similar but upon using looped inversions for the HMT instead of a CW saturation pulse. Parameters were similar as in Supplementary Figure 1: cross-relaxation rates were calculated for internuclear distance of $r = 2$ Å at 14.1 T. Relaxation rates were chosen to be $T_{2_l} = T_{2_{nl}} = 0.3\ s$, $T_{1_l} = 0.5\ s$ and $T_{1_{nl}} = 0.8\ s$, while water relaxation constants were taken to be $T_{2_w} = 0.5\ s$ and $T_{1_w} = 3\ s$.

In general, HMT enhancements will be determined not only by the chemical exchange rate, but also by the absolute values of the self- and cross-relaxation rates. Small cross-relaxation rates and fast rates of chemical exchange will significantly impair the efficiency of NOESY experiments, yet these are the scenarios where the fullest potential of MT encoded measurements arises. Supplementary Figure 2 illustrates the achievable enhancements that HMT can provide with respect to various rotational correlation times and chemical exchange rates, when normalized against conventional NOESY cross-peaks. Supplementary Figure 2a shows these enhancements assuming a continuous saturating RF with nutation field $\nu_1 = 20\ Hz$. Notice that enhancements show a non-monotonic behavior with respect to correlation times, which reflects the similar non-monotonic dependencies exhibited by the cross-relaxation rates (Supplementary Fig 2a, inset). Supplementary Figure 2b show a similar plot but for Hadamard MT achieved by looped inversion schemes, confirming very similar maximal enhancements. Notice that as solvent exchange rates become faster both approaches exhibit increases in their maximal enhancements, leading to ≥10-fold magnifications. Since these substantial enhancements are maximized when cross-relaxation rates are weak, it can be anticipated that they will not only arise from short correlation times but also for large internuclear distances of the kind that make dipole-dipole interactions weak. This provides HMT with an opportunity to reveal previously undetectable cross-peaks in challenging systems.

Further insight onto these joint correlation time / internuclear distances effects are presented in Supplementary Figure 3, calculated under the assumption of a continuous-wave

saturation. The magnitude of the normalized transferred magnetization is shown in Supplementary Figure 3a, plotted vs correlation times and internuclear distances for a water chemical exchange rate of 40 s^{-1}. As expected, the strongest cross-peaks can then be detected for slow tumbling times and short internuclear distances that make the cross-relaxation rates the strongest. By contrast, the HMT *enhancements* vs a conventional measurement (Supplementary Fig. 3b) show the opposite behavior: they are the smallest for strongest cross-relaxation rates and vice-versa. For this instance, for this particular exchange rate, HMT enhancements are >14-fold regardless of their means of encoding; thus, a conventional experiment would require almost 200 times longer acquisitions to achieve similar SNR –even before considering other sources of signal loss in the time-domain encoding. Moreover, if one arbitrarily defines 0.5% of the total magnetization intensity as an arbitrary experimental detection threshold, Supplementary Figure 4a provides an estimate of the range of distances that HMT could target. Assuming a medium-sized biomolecule with correlation time ~3 ns this cross-peak intensity threshold would provide correlations with the labile ^{1}H of up to 5.2 Å; in a fast tumbling regime where correlation times are ~0.1 ns, this distance drops to 3.5 Å. These figures are to be compared against what similar thresholds and models predict for a conventional NOESY experiment: 3.6 and 2.5 Å, respectively for each particular case.

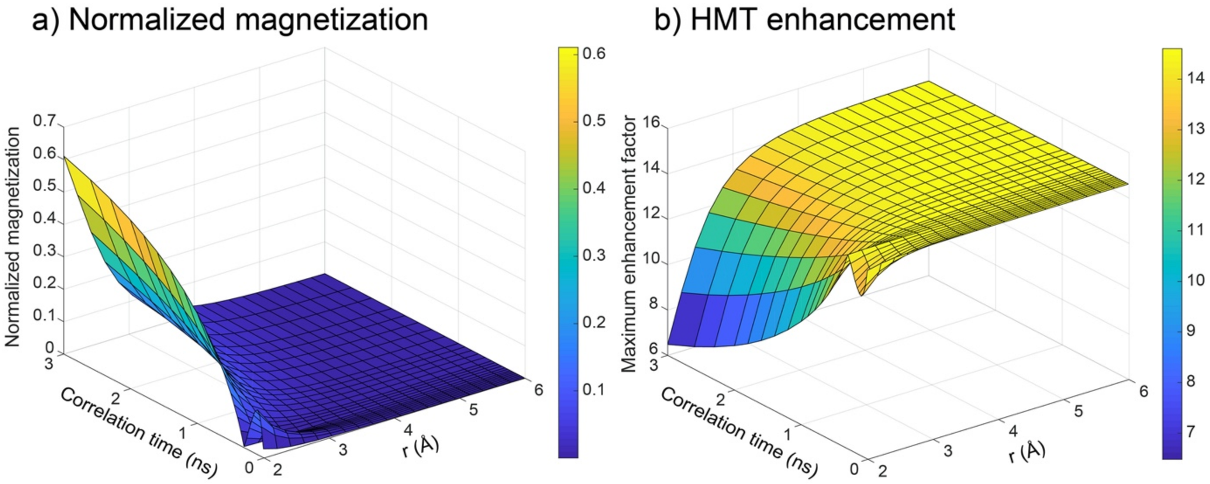

Supplementary Figure 3. Magnetization transferred in CW-based experiments. (a) Normalized magnetization transferred by continuous RF saturation from labile to non-labile protons, plotted with respect to correlation times and internuclear distances. (b) When these magnetization values are divided by the expectations for conventional NOESY cross-peaks, the HMT enhancements show an "anticorrelation" vs the surface plot in (a). A fast exchange rate of 40 s^{-1} was used in these simulations.

Supplementary Equations (11), used to estimate the efficiency of a looped inversion MT experiments, can also be used to estimate the enhancements achievable in HMT-based versions of the TOCSY experiment. As TOCSY requires a constant spin-lock to enable transfers through *J*-couplings, it follows that only such looped inversions (followed by isotropic mixing sequences) are compatible with the HMT schemes. To estimate the resulting enhancements, we replaced the cross-relaxation rate connecting non-labile and labile proton pools in Supplementary Eqs. (11), with an average *J*-coupling-based transfer rate, and the T_1 values appearing in these Eqs. with the spin-lattice relaxation times in the rotating frame ($T_{1\rho}$). Supplementary Figure 4 shows the expected enhancements in HMT versions of the TOCSY experiment, for two different coupling transfer rates: 7 Hz, representing a typical three-bond

coupling values, and 2 Hz, representing a longer-range proton-proton coupling. Notice that these HMT TOCSY experiments end up achieving somewhat smaller enhancements than their NOESY counterparts; this is due to the faster relaxation process (i.e., $T_{1\rho} < T_1$), constraining the extent of the transfer. Notice as well that higher enhancements are obtained for smaller values in the *J*-coupling (Supplementary Figure 4b), reflecting the fact that the chemical exchange involved will average out these weaker couplings, and hence further reduce SNR in conventional TOCSY transfers than in their looped counterparts.

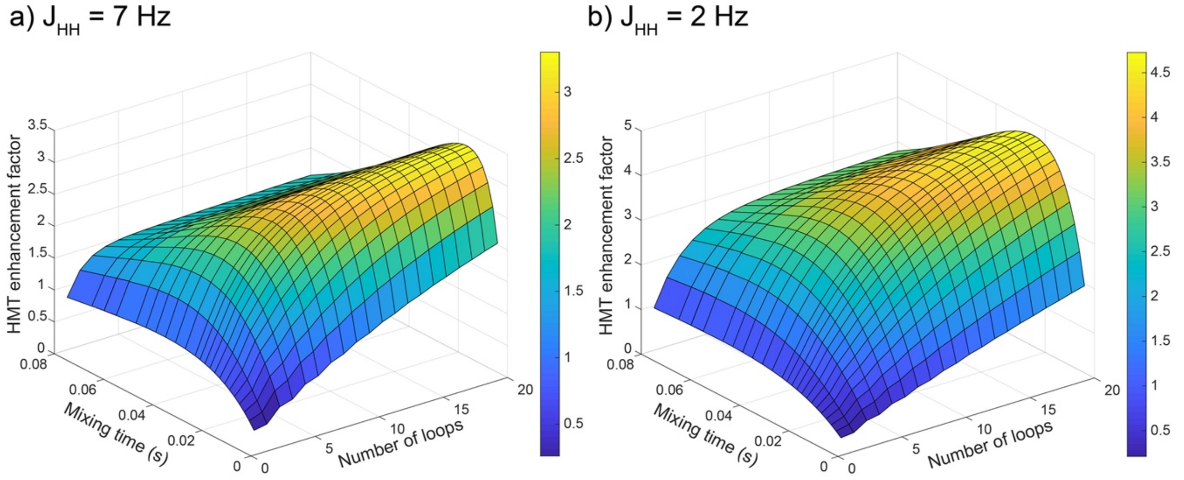

Supplementary Figure 4. Enhancements achievable in Hadamard MT TOCSY experiments comparing to conventional experiments for water exchange rate of 40 s^{-1} and two different *J*-coupling rates (a) 7 Hz which is typical ^{3}J coupling constant; (b) 2 Hz representing long range coupling. Relaxation rates were $T_{1\rho_l} = T_{1\rho_{nl}} = 0.3\ s$, and $T_{1\rho_w} = 0.5\ s$

HMT vs L-PROSY vs Conventional 2D TOCSY/NOESY.

HMT's principles are related to those underlying L-PROSY. We consider it therefore relevant to compare the L-PROSY experiment with HMT and conventional acquisitions. Supplementary Figure 5a illustrates 2D TOCSY NMR collected with all these methods on a 5 mM sucrose sample at 2 ºC. L-PROSY yields ≈2-4x sensitivity enhancements in its cross-peaks compared to conventional experiments; HMT yields similar enhancements for every cross peak. Supplementary Figure 5b shows another comparison for a NOESY experiment acquired on 5 mM myo-inositol; again, notice the ≈8-fold enhancements arising upon comparing L-PROSY and HMT with conventional NOESY, and the resemblance between the first of these two sets in terms of enhanced diagonal- and cross-peak SNRs. In both cases, however, the Hadamard encoding required acquisitions that were shorter by an order of magnitude than its Fourier-encoded counterparts, leading to a much better SNR/unit_time performance. This advantage is applicable if, as in all forms of 2D Hadamard NMR spectroscopy,[8,9] peaks are sufficiently resolved and their position *a priori* known.

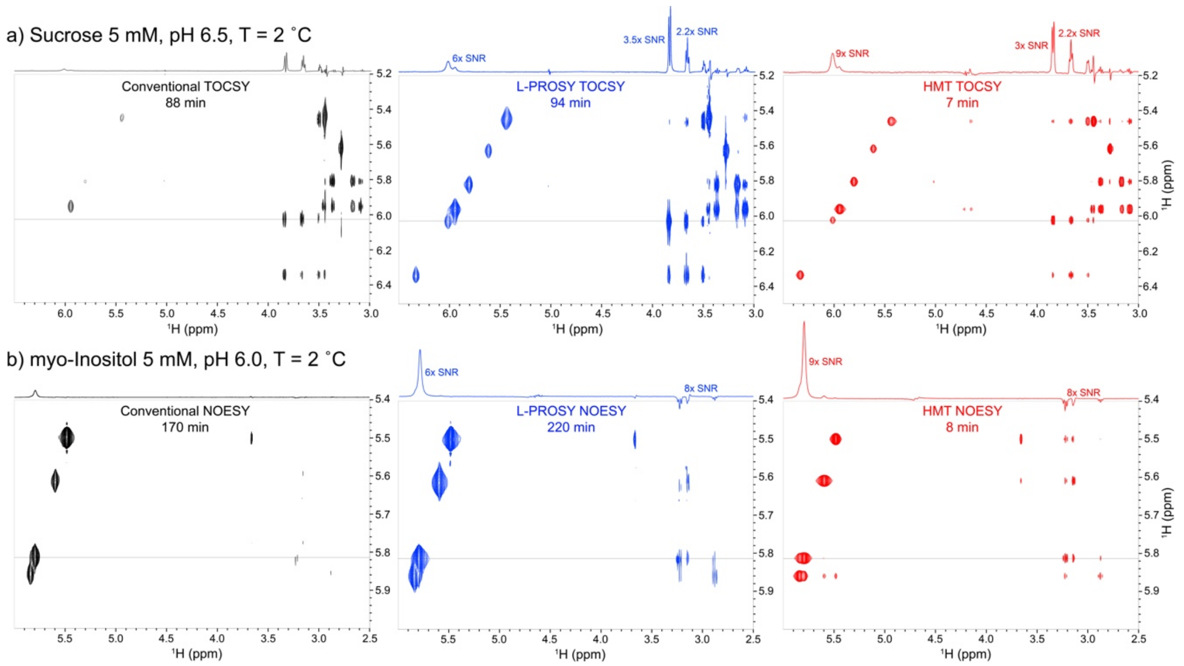

Supplementary Figure 5. HMT applied to simple sugars. a) TOCSY spectra acquired on 5 mM sucrose using conventional (48 ms DIPSI2), L-PROSY (11x20 ms DIPSI2) and Hadamard MT TOCSY (12x20 ms DIPSI2) schemes. Shown on top of each 2D spectrum are 1D traces extracted at the indicated horizonal dotted lines, with numbers indicating SNR improvements vs the conventional spectrum. Notice the superior spectral quality in both the L-PROSY and MT Hadamard experiments vis-à-vis the conventional TOCSY acquisition. b) Similar comparison but for NOESY experiments on 5 mM myo-Inositol. The conventional NOESY experiment was acquired with 80 ms mixing, L-PROSY used 14 loops, 35 ms each; Hadamard MT was acquired using 800 ms CW saturation. All data were acquired on a 600 MHz Avance III Bruker equipped with Prodigy probe.

HMT 2D TOCSY on oligosaccharides.

Supplementary Figure 6a compares a conventional NOESY collected using non-uniform sampling, and its HMT NOESY counterpart collected on $(SiA)_4$. These sets are equivalent to the data shown in Figure 2 of the main text, only acquired at 600 MHz instead of at 1 GHz. Notice that while a 14.1 T field is not high enough to resolve all hydroxyl resonances in this glycan, HMT still brings very large enhancements compared to its conventional NOESY counterpart. As further complement to the data shown in Figure 2 of the main text, Supplementary Figure 6b compares conventional and HMT-based TOCSY experiments recorded on $(Sia)_4$. Notice the superior quality and information content provided by the HMT experiment despite its ≥30-fold shorter acquisition time than that of the conventional experiment. In this case, the HMT TOCSY yields correlations with labile protons that are up to 5 bonds away (i.e. IOH7-I9), despite the presence of >100 s^{-1} chemical exchange with water.

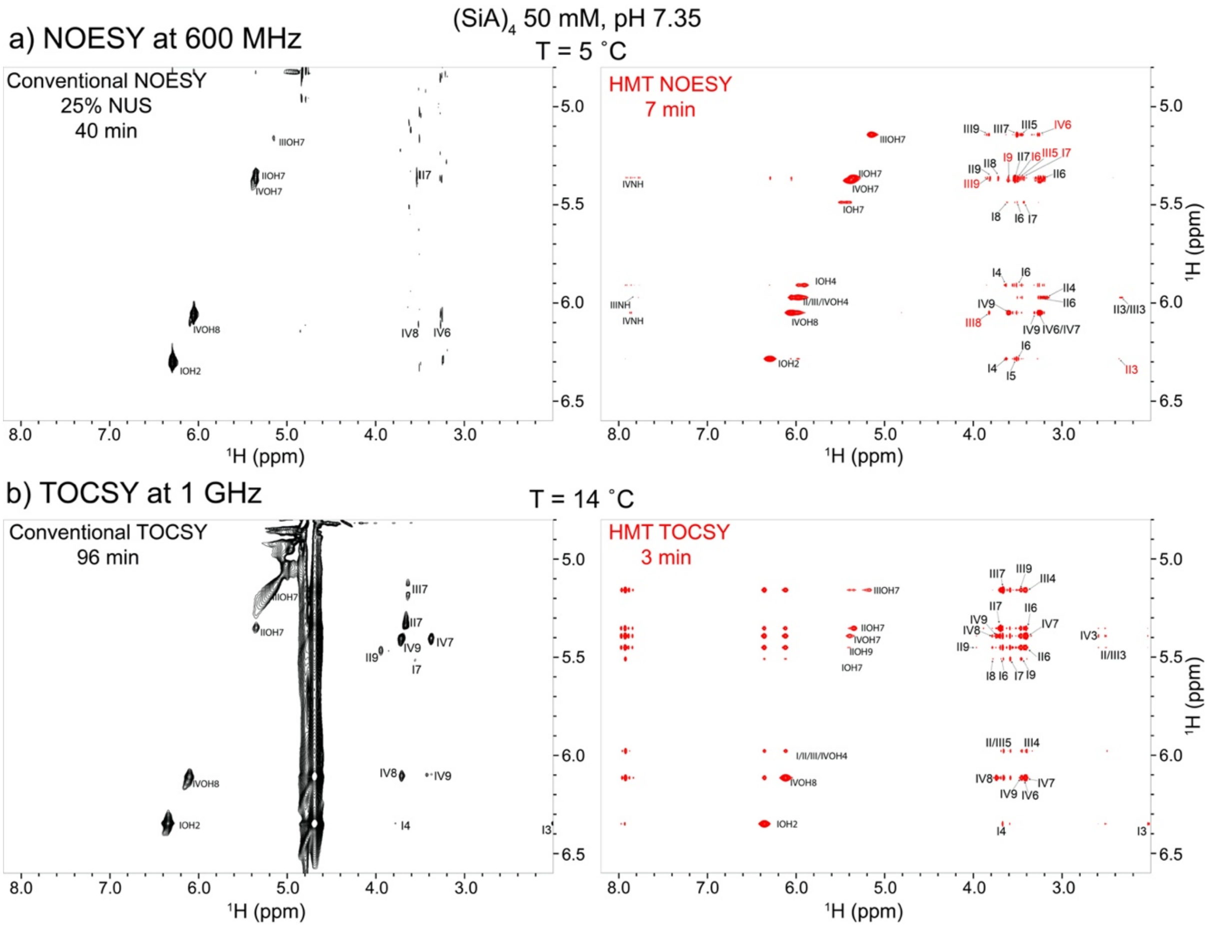

Supplementary Figure 6. HMT applied on a tetraglycan. (a) Comparison between non-uniformly sampled (NUS) Fourier and HMT NOESY spectra acquired on a $(SiA)_4$ sample at 600 MHz. (b) TOCSY spectra acquired on a 50 mM $(SiA)_4$ sample using conventional (40 ms DIPSI2) and Hadamard MT TOCSY (16x12 ms DIPSI2) at 1 GHz. Notice that while chemical exchange averages out *J*-couplings and cross-relaxation peaks rendering conventional TOCSY and NOESY experiments very inefficient, HMT provides numerous cross-peaks that help in both peak assignments and structure determination –despite requiring only a fraction of either the conventionally or non-uniformly sampled acquisition times.

HMT: Targeting "invisible" hydroxyl and amino correlations.

Complementing the HMT data in Figures 5a and 6b in the main text, Supplementary Figure 7a shows ubiquitin's Watergate 3919[10–12] spectrum highlighting the 6-10 ppm ^{1}H region, and the same spectrum after suppression of all ^{15}N/^{13}C bound protons[13–15] so as to reveal potential hydroxyl signatures. After examining these peaks at different temperatures (Supplementary Fig. 7b), it is possible to tentatively assign as hydroxyl ^{1}Hs those peaks that become sharper at lower temperatures; all the amide, amino and aromatic peaks are suppressed by multiple quantum filter, while the peaks that remain unchanged with temperature are attributed to unlabeled impurities present in the sample. Supplementary Figure 7c shows NOESY spectrum from Figure 4b in the main text zoomed on amino ^{1}Hs region showing exchange cross-peaks with another set of amines and some correlations to aliphatic ^{1}Hs region. Supplementary Figure 7d similarly shows hydroxyl ^{1}Hs region of conventional NOESY spectrum but acquired after suppression of ^{15}N/^{13}C-bound protons compared to HMT spectrum in Figure 6b.

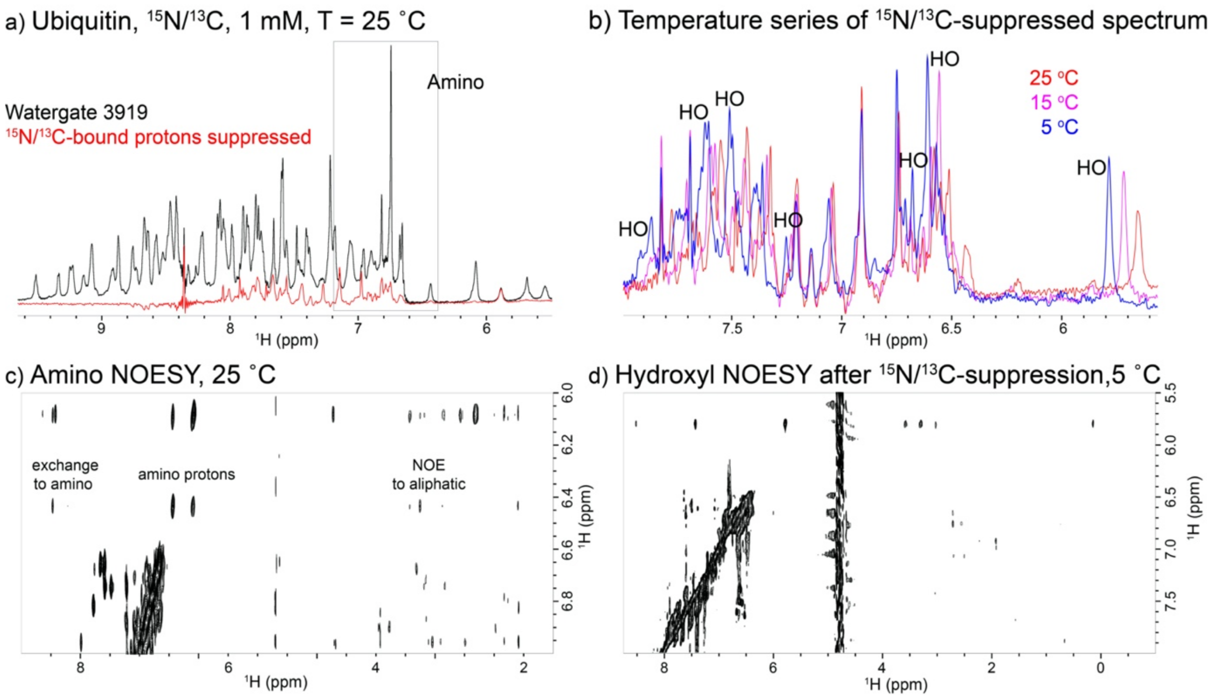

Supplementary Figure 7. Targeting "invisible" hydroxyl and amino ^{1}Hs by HMT. (a) Part of a ^{15}N/^{13}C decoupled WATERGATE 3919 spectrum of Ubiquitin illustrating amide and amine protons (black) at 25 °C and what remains from it after suppression of ^{15}N/^{13}C-bound protons. Shown by the dashed rectangle is the amino resonances region. (b) Temperature series of corresponding suppressed spectrum revealing peaks that are getting sharper at lower temperatures as potential hydroxyl protons. (c) Part of NOESY spectrum from Figure 6b zoomed on amino region. d) NOESY spectrum acquired after suppression of ^{15}N/^{13}C-bound protons using mixing time of 150 ms with total acquisition time of 4 hours.

In an extension of these measurements to the 14mer hairpin RNA, Supplementary Figure 8 presents a conventional full NOESY counterpart of the HMT NOESY data presented in Figure 6c of the main text, concentrating on the regions where amino (Supplementary Fig. 8b) and hydroxyl (Supplementary Fig. 8c) ^{1}Hs resonate. While amino ^{1}Hs show cross-peaks to imino and to other amino ^{1}Hs, correlations involving hydroxyl ^{1}Hs are harder to discern because they overlap with the amino resonances. The intrinsically selective nature of Hadamard encoding helps target these specific protons, and in combination with multiple quantum filter for ^{15}N/^{13}C-bound ^{1}H suppression, it yields correlations involving solely hydroxyl or amino ^{1}Hs (main text, Figure 6) that are clearly absent in Supplementary Fig. 8.

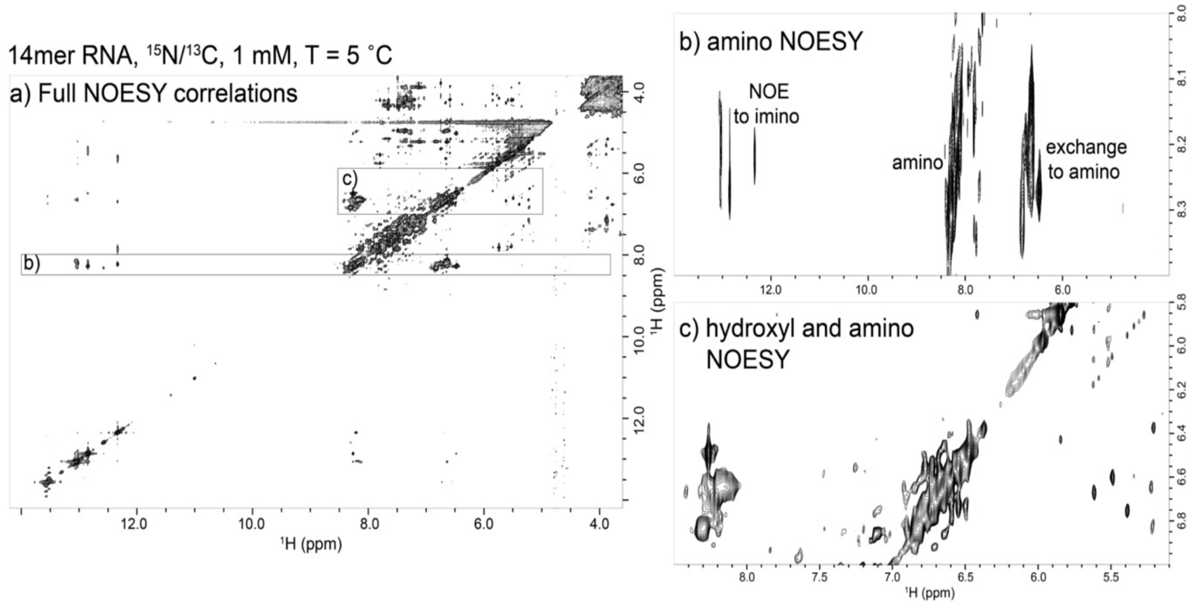

Supplementary Figure 8. Idem as Supp Figure 7 – but for RNAs. (a) Full NOESY spectrum acquired on 14mer hairpin RNA using 100 ms mixing and 90 us delay for a binomial water suppression. (b) Zoomed amino region of NOESY spectrum illustrating exchange correlations to amino protons and NOE cross-peaks to imino ^{1}Hs. (c) Portion of NOESY spectrum containing hydroxyl ^{1}Hs that overlap with amino resonances. Notice how difficult it is to discern these peaks from amino correlations in conventional spectra, when there is no suppression of ^{15}N/^{13}C-bound ^{1}Hs.

Supplementary Information – Literature References

1. McConnell, H. M. Reaction Rates by Nuclear Magnetic Resonance. *J. Chem. Phys.* **28**, 430–431 (1958).
2. Zhou, J. & Zijl, P. C. M. van. Chemical exchange saturation transfer imaging and spectroscopy. *Prog. Nucl. Magn. Reson. Spectrosc.* **48**, 109–136 (2006).
3. Helgstrand, M., Hard, T. & Allard, P. Simulations of NMR pulse sequences during equilibrium and non-equilibrium chemical exchange. *J. Biomol. NMR* **18**, 49–63 (2000).
4. Zaiss, M. & Bachert, P. Chemical exchange saturation transfer (CEST) and MR Z-spectroscopy in vivo: a review of theoretical approaches and methods. *Phys. Med. Biol* **58**, 221–269 (2013).
5. Novakovic, M., Cousin, S. F., Jaroszewicz, M. J., Rosenzweig, R. & Frydman, L. Looped-PROjected SpectroscopY (L-PROSY): A simple approach to enhance backbone/sidechain cross-peaks in 1H NMR. *J. Magn. Reson.* **294**, 169–180 (2018).
6. Novakovic, M., Martinho, R. P., Olsen, G. L., Lustig, M. S. & Frydman, L. Sensitivity-enhanced detection of non-labile proton and carbon NMR spectra on water resonances. *Phys. Chem. Chem. Phys.* **20**, 56–62 (2018).
7. Levitt, M. . *Spin Dynamics: Basics of Nuclear Magnetic Resonance*. *John Wiley & Sons Ltd* (2000). doi:10.1002/cmr.a.20130
8. Kupče, E. & Freeman, R. Frequency-domain Hadamard spectroscopy. *J. Magn. Reson.* **162**, 158–165 (2003).
9. Kupče, Ē. & Freeman, R. Two-dimensional Hadamard spectroscopy. *J. Magn. Reson.* **162**, 300–310 (2003).
10. Davis, A. L. & Wimperis, S. A solvent suppression technique giving NMR spectra with minimal amplitude and phase distortion. *J. Magn. Reson.* **84**, 620–626 (1989).
11. Piotto, M., Saudek, V. & Sklenář, V. Gradient-tailored excitation for single-quantum

NMR spectroscopy of aqueous solutions. *J. Biomol. NMR* **2**, 661–665 (1992).
12. Lippens, G., Dhalluin, C. & Wieruszeski, J. M. Use of a water flip-back pulse in the homonuclear NOESY experiment. *J. Biomol. NMR* **5**, 327–331 (1995).
13. Ogura, K., Terasawa, H. & Inagaki, F. An improved double-tuned and isotope-filtered pulse scheme based on a pulsed field gradient and a wide-band inversion shaped pulse. *J. Biomol. NMR* **8**, 492–498 (1996).
14. Zwahlen, C. *et al.* Methods for measurement of intermolecular NOEs by multinuclear NMR spectroscopy: Application to a bacteriophage λ N-peptide/boxB RNA complex. *J. Am. Chem. Soc.* **119**, 6711–6721 (1997).
15. Liepinsh, E., Otting, G. & Wüthrich, K. NMR spectroscopy of hydroxyl protons in aqueous solutions of peptides and proteins. *J. Biomol. NMR* **2**, 447–465 (1992).
